# DSN-STC: Leveraging Siamese networks for optimized short text clustering

**DOI:** 10.1371/journal.pone.0335709

**Published:** 2026-01-02

**Authors:** Mahdi Molaei, Mohammad-Reza Feizi-Derakhshi, Mohammad-Ali Balafar, Jafar Tanha

**Affiliations:** 1 Computerized Intelligence Systems Laboratory, Department of Computer Engineering, University of Tabriz, Tabriz, Iran; 2 Department of Computer Engineering, University of Tabriz, Tabriz, Iran; Zhejiang Normal University, CHINA

## Abstract

In this paper, we present a novel deep Siamese network with a multi-scale hybrid feature extraction architecture, named DSN-STC (Deep Siamese Network for Short Text Clustering), that significantly improves the clustering of short text. A key innovation of our approach is a specialized transformation mechanism that maps pre-trained word embeddings into cluster-aware text representations. In this new latent space, the proposed model minimizes the overall overlapping between clusters while improving the cohesion within each cluster. This results in considerable improvements in clustering performance. Since short texts inherently contain both sequential context and localized patterns within their limited context, in this paper a hybrid approach is used by combining both recurrent layers and multi-scale convolutional neural networks to maximize the extractable feature sets from their limited context. This architecture allows us to capture the sequential features and local dependencies by recurrent layer and convolutional layers respectively which leads to generating a more accurate and rich representation for each short text. To evaluate our architecture and because our main focus is on clustering Persian short text, several experiments are conducted in which the results show that the DSN-STC outperforms other approaches in clustering accuracy (ACC) and normalized mutual information (NMI) metrics. Also to further test the proposed architecture’s generalizability and adaptability in other languages, DSN-STC is evaluated on 2 English benchmark datasets where it consistently outperformed previous approaches in both metrics. These results highlight the model’s ability to learn robust and cluster-aware feature representations that are highly useful for effective short text clustering.

## Introduction

The exponential growth of textual documents available on the Internet in recent years has significantly increased, especially with the advent of social media platforms. With mobile devices and Internet technologies advancing quickly, users have become motivated to search for information, communicate with their peers, and share opinions and thoughts on social media platforms like Twitter, Instagram, Facebook and search engines such as Google. The sheer amount of short text generated daily from these platforms leads to a large volume of overall unstructured data.

Short texts are characterized by their brevity, often lacking sufficient context, making knowledge extraction challenging. Despite their brevity, short texts are rich in information and play a crucial role in numerous applications, including information retrieval, sentiment analysis, and topic detection. However, their unstructured nature presents significant challenges for data analysis, particularly in clustering, where the goal is to automatically identify valuable patterns within large collections of text [1].

Among the various data-mining techniques, clustering stands out as an essential method for analyzing short text corpora. Clustering short texts can be highly beneficial across diverse fields like information retrieval. For example, clustering can group similar short texts in an unsupervised manner, facilitating tasks such as topic detection in short news texts. This automation reduces the need for human intervention, saving time, cost, and resources, which are typically required for manual labeling. However, clustering short texts is particularly challenging due to their chaotic nature, often containing noise, slang, emojis, misspellings, abbreviations, and grammatical errors [1]. Additionally, the short length of these texts exacerbates issues related to data sparsity, limited context, and high-dimensional representation. Standard clustering techniques, such as k-means [2] or DBSCAN [3], often struggle to accurately group short texts because these methods rely heavily on measuring similarity or distance between data points and depend on accurate text representations [4]. When applied directly to short text corpora, traditional techniques tend to perform poorly, as the sparse and high-dimensional feature vectors generated by standard text representation methods like term frequency-inverse document frequency (TF-IDF) or bag of words (BoW) [5] are less effective in capturing meaningful distances between data points [6].

To address these challenges, dimensionality reduction is often employed as an essential step in the short text clustering (STC) process. One notable advancement in this area was the introduction of Deep Embedded Clustering (DEC) [7], which utilized an autoencoder (AE) network for dimensionality reduction before applying k-means clustering. This approach marked a significant step forward in tackling the high-dimensionality problem inherent in short text representation. However, despite these advancements, significant challenges persist. One notable issue is that the dimensionality reduction achieved through AE networks can inadvertently increase overlap between clusters, potentially degrading the overall clustering performance.

Recognizing the limitations of existing single‐branch and purely unsupervised schemes, this paper introduces a sophisticated Siamese‐based architecture that jointly tackles both dimensionality reduction and cluster separability within a unified training framework. Siamese networks, first introduced in 1994, are typically used for tasks involving similarity detection [8]. These networks consist of two identical subnetworks with shared parameters, which are trained simultaneously to generate outputs for two input instances. The network then updates its weights based on whether the two inputs belong to the same class, aiming to reduce the distance between embeddings of similar texts and increase the separation between those of different classes by a margin *m*. To advance this paradigm, our proposed DSN-STC integrates a hybrid feature extraction framework that jointly combines two complementary branches: a recurrent neural network to model long-range contextual dependencies and a multi-scale convolutional module to capture local n-gram patterns across varying granularities. The extractable features from the limited context of short text are maximized by this architecture.

Beyond its architectural innovations, DSN-STC learns a mapping from word embeddings into text representations $f_{\theta }:R^{D}\to R^{d}$ that projects high-dimensional input vectors into a dense, cluster-aware latent space. The contrastive loss not only ensures tight intra-cluster cohesion and wide inter-cluster gaps, but also implicitly encourages the network to emphasize features with strong cluster-label correlations. Theoretically, this encourages the network to identify and amplify key lexical or syntactic features such as domain-specific keywords or phrase structures, that carry the highest mutual information with respect to cluster labels. In this way, DSN‐STC not only reduces representational dimensionality but also systematically mitigates cluster overlap thereby resolving two of the principal challenges identified in prior short-text clustering research.

In addition, we observed that texts with fewer than 10 tokens, after preprocessing, often lack sufficient information to be encoded into meaningful representations for clustering purposes. As a result, the dataset is filtered to include only texts with a length between 10 and 30 tokens. This range was chosen to focus on short texts while still retaining enough content for effective clustering. By filtering out extremely short texts, it is ensured that our method could produce more meaningful representations, leading to more accurate clustering results. Furthermore, although this study concentrates on Persian short texts, the DSN-STC was also evaluated on English corpora and found to yield similarly strong gains. This cross‐linguistic evaluation confirms that our architecture is not tied to a single language’s characteristics but generalizes robustly across different linguistic settings.

Our extensive evaluations demonstrate that the proposed approach significantly outperforms previous methods, achieving superior clustering performance.

The main contributions of this paper are:

1. **Contrastive Siamese Pre-trained Word Embeddings Transformer for Learning Cluster-Aware Text Representations**: A novel Siamese‐network architecture has been formulated to learn dense, cluster‐aware representations for short texts. By jointly optimizing a contrastive loss, the model provably minimizes intra‐cluster cohesion while maximizing inter‐cluster margins in the new latent space. This theoretical design ensures that high‐dimensional, sparse text inputs are mapped into a lower‐dimensional latent space where cluster overlap is rigorously controlled, leading to more separable and robust clusters. Evaluation results confirm that the learned margin satisfies bounds on cluster cohesion and separation, extending prior work on contrastive representation learning.
2. **Multi-Scale Recurrent–Convolutional Fusion for Rich Text Representations**: A novel hybrid architecture is presented that fuses bidirectional sequential features with multi-scale n-gram dependencies through parallel processing paths. In this design, one path employs recurrent units to model long-range contextual interactions, while the other leverages convolutional filters of varying kernel sizes to extract localized n-gram patterns at multiple granularities. This higher-dimensional basis enables the model to implicitly identify and amplify those token sequences or phrase structures that carry maximal mutual information and can reflect the subject of text better. In addition these rich representations are further refined via contrastive training, the resulting the new latent space becomes both dense and highly discriminative. Empirically, this synergy between long-range dependency modeling and localized pattern detection yields a more informative representation space and leads to significant improvements in clustering accuracy.
3. **Focus on Effective Text Length for Clustering**: An optimal token‐count window of 10–30 was determined and applied to ensure that each document contains enough semantic information while excluding outliers, excessively short texts lacking context or overly long passages that may introduce noise. Texts outside this range were removed, resulting in a corpus of concise yet contextually rich inputs. This filtering strategy produced more informative representation and yielded consistent gains in clustering accuracy and cluster coherence which is shown in experiments.

The remainder of this paper is organized as follows. The Related Work section surveys prior work in short-text clustering, grouping methods into several categories and highlights the gaps that our DSN-STC addresses. The Methods section details the proposed DSN-STC architecture, including its Siamese network design, multi-scale hybrid feature extraction architecture, and contrastive-loss training procedure. The Experiments section describes our experimental setup including datasets, preprocessing, hyperparameters (Table 1), evaluation metrics, and present results across empirical evaluations, ablation studies, statistical validation, and cross-linguistic tests. The Discussion section discusses key findings, strengths, and limitations of DSN-STC. Finally, the Conclusions and Future Works section concludes the paper and outlines directions for future work.

**Table 1:** Hyperparameter settings for DSN-STC.

Parameter	Value
Random seed	73
Test Split	0.2
Validation Split	0.2
Batch Size	256
Epochs	200
EarlyStopping	Monitor val_loss with patience = 10
Optimizer	Adam
Activation Function	ReLU
Recurrent layer units	200
Conv1D filters (kernel sizes 3, 5, 7)	64 each
FC1 & FC4 units	100
FC2 & FC3 units	50

### Related work

The field of short‐text clustering has evolved rapidly, encompassing a variety of deep‐learning architectures and representation strategies. This section reviews four key categories of prior work, including deep embedded clustering, contrastive and Siamese approaches, transformer‐based clustering, and hybrid recurrent–convolutional models, and then highlights how DSN-STC advances beyond each.

Deep Embedded Clustering (DEC) was one of the pioneering approaches to introduce a deep learning-based method in the field of clustering [7]. DEC uses an autoencoder (AE) network to extract feature representations, which are crucial for overcoming the limitations of traditional clustering techniques that struggle with high-dimensional and sparse data, such as short texts. In this approach, feature representation learning and clustering are performed jointly by combining the autoencoder with the k-means algorithm. This approach demonstrated superior performance, outperforming previous methods in both image and textual data clustering tasks. Following the introduction of Deep Embedded Clustering (DEC), many classic methods have been proposed for the advancement of the deep learning-based text clustering field. Examples include Improved Deep Embedded Clustering (IDEC) [9], Short Text Clustering with SIF Embeddings (STC) [10], Deep Clustering Network (DCN) [11], and DEC with Data Augmentation (DEC-DA) [12], each of which introduce their own methodology for improving the original method, focusing on different components of the clustering method. These approaches share a common thread of jointly learning feature representations and cluster assignments, but they differ in their specific implementations and each of these methods aimed to address specific challenges in clustering tasks, such as better handling of data sparsity, improving feature representation, or enhancing the separation between clusters. These models consistently demonstrated improved performance across various datasets by introducing more sophisticated techniques for joint learning of feature representations and cluster assignments. They showed notable success in clustering both image and textual data, further advancing the field of deep learning-based clustering methods. Self-Taught Convolutional Neural Networks for Short Text Clustering also addresses the sparsity of short‐text by first compressing raw features into binary codes, then training a CNN to fit those codes while learning semantic representations, and finally applying K-means on the learned features [4]. This framework demonstrated that unsupervised CNNs, guided by auxiliary codes, can effectively capture both local n-gram patterns and global semantic structure in brief texts.

In the realm of deep learning-based clustering, a notable advancement came with the introduction of a novel approach that leverages deep neural networks to simultaneously learn feature representations and suitable embeddings [13]. This method builds upon the foundation laid by earlier techniques like DEC [7]. At its core, the approach utilizes an autoencoder for dimensionality reduction, followed by a specialized representation network connected to the encoder’s output. The key innovation lies in its objective to maximize inter-cluster distances by minimizing cross-entropy between pairwise similarity distributions in the autoencoder’s latent space and the representation network’s embedding space. This strategy effectively encourages maximum separation between clusters, addressing a common challenge in clustering tasks. Evaluated on both textual and image datasets, the method demonstrated significant improvements over its predecessors, marking a step forward in the field of unsupervised learning and clustering. The paper [14] tackles the challenges of clustering sparse and high-dimensional short texts. The authors propose two methods using unsupervised autoencoders to enhance text representation. The first, Structural Text Network Graph Autoencoder (STN-GAE), combines text network structure with pre-trained features using graph convolutional networks. The second, Soft Cluster Assignment Autoencoder (SCA-AE), adds a soft clustering constraint in the latent space to improve clustering-aware representations. Experiments on seven datasets show significant improvements over traditional models, with the SCA-AE achieving up to 14% better accuracy compared to BERT. In [15] Guan et al. introduce a novel framework that overcomes the limitations of traditional text clustering methods. Recognizing the shortcomings of bag-of-words models in handling high dimensionality, sparsity, and sequential information, the authors propose a deep feature-based approach. The DFTC framework leverages pre-trained text encoders to capture rich semantic representations, reducing the reliance on supervised learning often associated with deep learning-based clustering. Empirical evaluations demonstrate DFTC’s superior performance over traditional methods and state-of-the-art models like BERT across diverse datasets. To enhance interpretability, the paper presents the Text Clustering Results Explanation (TCRE) model, which provides insights into the semantics of the formed clusters. This contribution significantly advances the field of text data analysis by offering both improved clustering accuracy and meaningful explanations. Ding and Mei, in [16] proposed a framework that incorporates semantic fusion into the BiLSTM-CNN architecture, which improves short text classification via a number of local and contextual features. Using the Skip-gram model to embed words, local features are captured through the CNN, while global context is handled by BiLSTM, which improves upon the limitations of more classical models. Multiple tests conducted on several datasets demonstrate method’s superior performance relative to other classification models and present a robust method that reclines upon a new method of feature extraction, which enhances performance when classifying short text. The paper [17] presents a novel method for clustering short texts, particularly from social media. The authors address challenges like data sparsity and non-standard language by integrating BERT, which captures contextual semantics, with the Biterm Topic Model (BTM) to analyze word co-occurrences and extract topics. Using the DBSCAN algorithm, the proposed approach demonstrates high clustering accuracy and improved text processing quality. This research offers a valuable contribution to short text analysis, especially in environments with sparse and variable data. Paper [18], tackle the issues of feature sparsity and semantic ambiguity often found in short texts. They introduce the DCAN model, which combines convolutional neural networks (CNN) with an attention mechanism and dynamic routing to improve the extraction and fusion of features. The model first uses CNN to capture features at various levels of granularity, enriching the text’s semantic representation. It then applies an attention mechanism to prioritize relevant features based on context, followed by dynamic routing to optimize information flow between layers. Tested on datasets like AG News and SST-2, DCAN demonstrated superior accuracy over existing methods, offering a valuable advancement in short text classification and natural language processing. Further advancements have focused on improving the robustness of these models by explicitly addressing noise and outliers in embedding-based clustering using a combination of Frobenius-based reconstruction with sparsity-promoting or elastic penalties and by incorporating graph-based regularizes to preserve local geometry [19,20]. These approaches improve robustness to Laplacian/outlier noise and enhance the topology of learned representations, which can be beneficial for sparse or noisy short-text corpora.

Other methods have explored hybrid deep–probabilistic models, such as combining autoencoder representations with Gaussian Mixture Models (GMMs) [21]. The authors design a framework that enables the joint optimization of both data representations and GMM parameters, which helps achieve more compact clusters and better separation between them. The process starts with an autoencoder extracting features from unlabeled data, which are then modeled by a GMM. What sets this method apart is its adaptive mechanism, where the GMM parameters are continuously refined based on the learned features, allowing the model to align more accurately with the true data structure. This twofold optimization not only captures the distribution more precisely but also enhances the clustering by ensuring the Gaussian components distinctly represent separate clusters. Tests on eight datasets show that the method surpasses several existing advanced clustering techniques, making it particularly effective for unsupervised learning. This contribution offers a robust solution that combines deep learning with probabilistic modeling to advance clustering techniques.

Siamese networks have ability to learn meaningful representations by analyzing the relationships between document pairs. These networks are particularly effective at improving clustering performance, especially when dealing with high-dimensional, sparse data. By focusing on the similarities and differences between texts, Siamese architectures allow for more nuanced and context-aware clustering results. In the paper [22], authors propose a deep Siamese neural network that addresses the shortcomings of traditional high-dimensional text representation methods. This model learns low-dimensional document embeddings by focusing on semantic similarities between documents, which improves text classification tasks. Using two sub-networks based on multi-layer perceptrons (MLPs), the network is trained to boost similarity scores for documents within the same category and reduce them for those in different categories. Tested on the BBC news dataset, the method significantly outperforms conventional approaches, offering important insights into improving text categorization with advanced neural architectures. The paper [23] proposed a model based on the Siamese Neural Network (SNN) which is used for calculating semantic similarities between different languages and domains. They improved classic SNNs using the ReLU activation function and lexical feature sets, leading to improvements in measuring semantics between short text pairs. The proposed method is evaluated on both English and Portuguese datasets. The results outperformed baseline models and showed proposed method’s effectiveness in cross-lingual and cross-domain text similarity tasks. This research offers a valuable approach to improving semantic similarity with minimal training data. A novel methodology to boost textual clustering by integrating semi-supervised learning approaches is proposed in [24]. The authors use pairwise constraints to guide the clustering process. These constraints specify whether pairs of documents should be grouped together (‘must-link’) or kept apart (‘cannot-link’). Pairwise clustering performance improved significantly. The method introduced is based on Convolutional Siamese Network (CSN) that can learn a low-dimensional representation of the documents, which captures semantic similarities between the documents. The low-dimensional representations are learned and optimized with the pairwise constraints, leading to improvement in the quality of the clusters. After the representations are learned, a K-Means algorithm is used to cluster the documents. The authors demonstrate performance on 8 data sets, and the proposed method shows consistent improved performance against alternative clustering methods, i.e., MPC-KMeans [25] and standard K-Means preferences. This paradigm has been most powerfully realized in recent years through the adoption of transformer architectures. Models such as Sentence-BERT (SBERT) have set a new standard by fine-tuning pre-trained language models on sentence-pair objectives, thereby producing embeddings that capture nuanced semantic relationships with unprecedented accuracy [26].

Many architectures for text clustering have been proposed, but relatively few have focused specifically on short texts. A fundamental challenge in these approaches is the generation of cluster-aware, low-dimensional representations that exactly encode the salient features of the original text while improving effective clustering. Although existing methods successfully integrate representation learning with clustering, they frequently employ single-branch networks or rely on unsupervised coding schemes that may inadequately capture cluster structure in short-text contexts. Transformer-based clustering solutions (e.g., BERT + K-means) provide strong baselines but incur substantial computational overhead. In contrast, the proposed method, DSN-STC, is constructed as a semi-supervised, multi-scale hybrid Siamese network combining recurrent and convolutional feature extractors which is trained with a contrastive loss to map initial embeddings into a cluster-aware space where representations are both lower in dimensionality and more distinctly separated. Consequently, inter-cluster distances are maximized and intra-cluster distances minimized which causes minimizing clusters’ overlap and more accurate clustering outcomes. This design has the potential to transform large-scale short-text analysis by producing embeddings that are inherently optimized for clustering tasks.

### Problem definition

Let $\left(x_{i},x_{j}\right)$ be an input pair, where $x_{i}$ and $x_{j}$ are the word embeddings of short text that are generated from the pre-trained word embedding model. Both $x_{i}$ and $x_{j}$ are fed into a shared neural network $f_{\theta }$, parameterized by θ, and then two feature vectors ${f_{\theta }(x}_{i})$ and ${f_{\theta }(x}_{j})$ are generated at the output:


$$h_{i}=f_{\theta }\left(x_{i}\right),\;\;h_{j}=f_{\theta }\left(x_{j}\right)$$ (1)

For contrastive supervision we constructed an exhaustive pairwise training set using the available class labels. Concretely, given N sentences ${\left\{x_{i}\right\}}_{i=\text{1}}^{N}$ with ground-truth labels ${\left\{c_{i}\right\}}_{i=\text{1}}^{N}$, we built the binary affinity matrix $A=\left[a_{ij}\right]\;\in {\left\{\text{0},\text{1}\right\}}^{N\times N}$ with:


$$a_{ij}=\;\left\{ \begin{array}{c} \text{1}\;\;\;\;\;if\;c_{i}=c_{j}\;\;\;\;\;(positive\;pairs)\\ \text{0}\;\;\;\;\;if\;c_{i}\neq c_{j}\;\;\;\;\;(negative\;pairs) \end{array}\right.\;$$ (2)

This procedure generates a complete training set of N×N labeled pairs, where every ordered pair $\left(x_{i},\;x_{j}\right)$ receives a corresponding label $A_{ij}$. We use these labeled pairs as the supervised signal for the Siamese contrastive objective: positive pairs are driven to small embedding distance while negative pairs are pushed to lie beyond the margin *m*. The exhaustive construction maximizes the available pairwise supervision and provides a dense, stable training signal for contrastive learning in our semi-supervised setup.

Instead of Euclidean distance, we measure similarity via the cosine distance:


$$d_{cos}\left(h_{i},h_{j}\right)=\text{1}-\frac{h_{i}\cdot h_{j}}{\left\|h_{i}\right\|\left\|h_{j}\right\|}$$ (3)

which lies in the interval [0,2] since cos(·)∈[−1,1]. In practice (explained later in the experimental setup section) encoder outputs are L2-normalized before computing cosine distance.

### Proposed method

This study did not involve human participants, identifiable human data, or animals; therefore, ethical approval and informed consent were not required.

**Fig 1:**
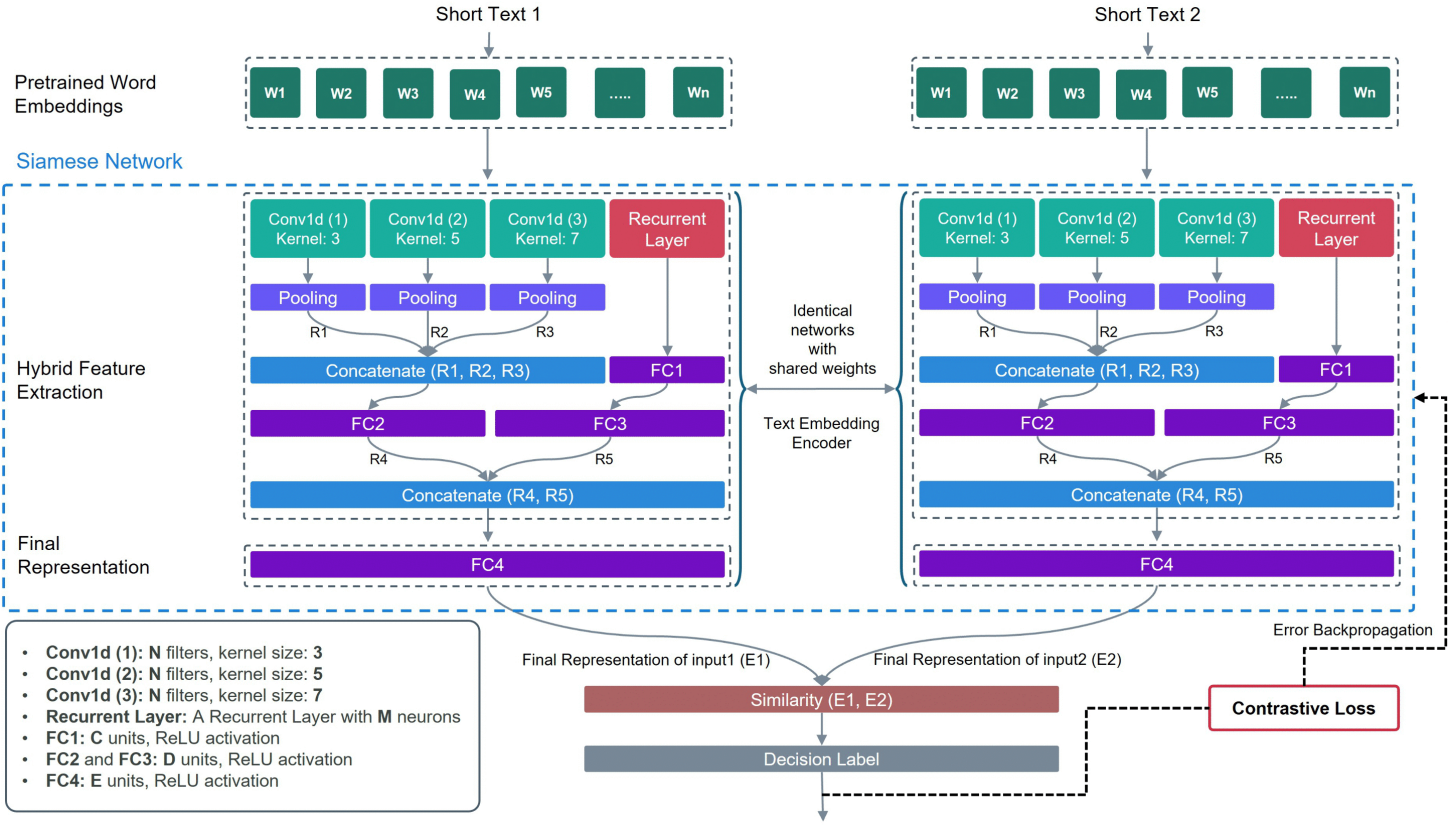
Proposed architecture of DSN-STC.

The overall architecture of DSN-STC is depicted in Fig 1. Each Siamese branch begins by feeding a sequence of word embeddings for two input texts. These embeddings are then processed by the hybrid feature extraction architecture, which consists of three parallel modules: a recurrent layer, three convolutional layers with kernel sizes 3, 5, and 7, and a stack of fully connected layers to extract multi-scale, cluster-aware features. Specifically:

1 Recurrent Path:

A recurrent layer models long-range dependencies and produces hidden states {ℎ_t_}. These states are projected through two dense layers to yield a dense feature vector $r\in R^{d_{r}}$.

2 Convolutional Path:

Three 1D convolutions capture local n-gram patterns at varying granularities. Their outputs are concatenated and passed through a dense layer to form $C\in R^{d_{c}}$.

3 Fusion and Projection:

The vectors r and c are concatenated and mapped by a last dense layer into the final representation $z=f_{\theta }(x)\in R^{d}$. A contrastive-loss head then enforces pulling together similar pairs and pushing apart others by margin m.

Theoretically, this design learns a mapping from word embeddings into text representations $f_{\theta }:R^{D}\to R^{d}$ into a cluster‐aware latent space, where representations are both lower in dimension and more separable. Each sentence contains both local and long-range dependencies among its words. For example, consider this sentence: “Alice, who had traveled the world, cherishes her childhood memories.” Capturing and extracting local patterns (e.g., “childhood memories” in this example) requires convolutional layers, while modeling the relation between “Alice” and “cherishes” demands long-range context which can be extracted by the recurrent layer. This combination of convolutional and recurrent features equips the network with a multi-faceted latent space in which the contrastive loss can more effectively discern and reinforce cluster structure. Furthermore, by emphasizing dimensions that capture both salient local patterns and long-range relational context, intra-cluster cohesion is strengthened, and inter-cluster separation is enlarged. Consequently, these representations become inherently optimized for downstream clustering tasks. Detailed configurations for each module follow in the subsections below.

### The Siamese network

A Siamese neural network consists of two identical subnetworks that share the same weights, architecture, and parameters. These subnetworks process two inputs in parallel, which are then compared to determine their similarity or distance. Then the outputs, which are two final representations of input short text, are evaluated using the contrastive loss function that measures the distance between these two representations $\left(h_{i},\;h_{j}\right)$ and tries to maximize or minimize the existing distance between them based on their labels that show they are in the same cluster or not. The contrastive loss is defined as follows in (4):


$$L(h_{i},\;h_{j})=\frac{\text{1}}{\text{2}}[a_{ij}d_{cos}{\left(h_{i},\;h_{j}\right)}^{\text{2}}+\left(\text{1}-a_{ij}\right)\;\text{max}(\text{0},\;m-{d_{cos}(h_{i},\;h_{j}))}^{\text{2}}],\;m\in (\text{0},\;\text{2}]$$ (4)

where $d_{cos}$ is the cosine distance (Eq 3) measured between the embeddings, $a_{ij}$ is a binary label which is taken from affinity matrix A, ***m*** is a margin that defines the minimum distance between dissimilar pairs, ensuring that negative pairs are pushed apart by distance of *m.*

The overall loss $L_{batch}$ minimized for a training mini-batch *B* is the mean of the pairwise loss over all |*B*| pairs in the batch:


$$\begin{array}{c} L_{batch}=\;\frac{\text{1}}{\left|B\right|}\;\sum {}_{(i,\;j)\in B}L(h_{i},\;h_{j}) \end{array}$$ (5)

**Proposition 1: Margin-Separation Guarantee in Theory**

Assume encoder outputs are L2-normalized (as done in training). If training reaches perfect convergence on the training set (i.e., when L = 0 for all pairs), the model guarantees:

1 Cluster Cohesion: Every positive pair $\left(h_{i},h_{j}^{+}\right)$ is mapped to the same point, resulting in a cosine distance of zero:

$$d_{cos}\left(h_{i},h_{j}^{+}\right)=\text{0}$$

Therefore, the maximum intra-cluster distance is $\delta_{intra}=\text{0}$.

2 Cluster Separation: Every negative pair $\left(h_{i},h_{j}^{-}\right)$ is separated by a margin of at least m:

$$d_{cos}\left(h_{i},h_{j}^{-}\right)\geq m$$

Therefore, the minimum inter-cluster distance is at least *m*.

In other words, this is not an assumption but a direct result of convergence. With $\delta_{intra}=\text{0}$ and the minimum negative-pair distance being *m*, we have a guaranteed inter-cluster gap of at least *m* in cosine distance.

**Proof Sketch:**

The proof follows from the definition of the contrastive loss function. At the point of training convergence, the total loss *L* is zero. Since the loss function is a sum of non-negative terms, the loss contribution for every individual training pair must also be zero. We analyze the two cases:

1 Positive pairs $(a_{ij}=\text{1}):$

For a positive pair, the corresponding loss term is $\frac{\text{1}}{\text{2}}d_{cos}{\left(h_{i},h_{j}^{+}\right)}^{\text{2}}$. For the total loss to be zero, this term must be zero:


$$\frac{\text{1}}{\text{2}}d_{cos}{\left(h_{i},h_{j}^{+}\right)}^{\text{2}}=\text{0}\;\Rightarrow d_{cos}=\text{0}$$

This demonstrates that all positive pairs are mapped to the same point in the embedding space and have a cosine distance of zero.

2 Negative pairs $(a_{ij}=\text{0}):$

For a negative pair, the loss term is $\frac{\text{1}}{\text{2}}max{\left(\text{0},m-d_{cos}\left(h_{i},h_{j}^{-}\right)\right)}^{\text{2}}$. For this term to be zero, the argument of the squared max function must itself be zero:


$$\text{max}\left(\text{0},m-d_{\text{cos}}\left(h_{i},h_{j}^{-}\right)\right)=\text{0}$$

This equality holds only if the term inside is non-positive, which means $m-d_{cos}\left(h_{i},h_{j}^{-}\right)\leq \text{0}$. This directly implies:


$$d_{cos}\left(h_{i},h_{j}^{-}\right)\geq m$$

**Theoretical Bounds in the Non-Asymptotic Case:**

Beyond the idealized convergence guarantee presented in Proposition 1, the contrastive loss function offers a deeper theoretical strength by providing explicit bounds on cluster quality in the practical, non-asymptotic case. While the analysis above describes the ideal convergence scenario, in practice, the model converges to a state where the loss for any given pair is bounded by a small residual value, ε>0. In this more realistic scenario, the structure of the loss function allows us to derive formal guarantees on the final cluster structure:

- Bound on Cluster Cohesion: For any positive pair $\left(a_{ij}=\text{1}\right)$, the loss term is $L_{pos}=\frac{\text{1}}{\text{2}}d_{cos}^{\text{2}}$. If we assume $L_{pos}\leq \varepsilon$, it follows that $d_{cos}^{\text{2}}\leq \text{2}\varepsilon$, which provides an upper bound on the intra-cluster distance:

$$d_{\text{cos}}\leq \sqrt{\text{2}\varepsilon }$$

- Bound on Cluster Separation: Similarly, for any negative pair $\left(a_{ij}=\text{0}\right)$, the loss is $L_{neg}=\frac{\text{1}}{\text{2}}\text{max}(\text{0},\;m-d_{cos}{)}^{\text{2}}$. Bounding this loss by ε implies that $\text{max}(\text{0},\;m-d_{cos})\leq \sqrt{\text{2}\varepsilon }$. Since the *max* term must be non-negative, this simplifies to $\;m-d_{cos}\leq \sqrt{\text{2}\varepsilon }$, which provides a lower bound on the inter-cluster distance:

$$d_{\text{cos}}\geq m-\sqrt{\text{2}\varepsilon }$$

This analysis demonstrates that the quality of the final clustering, both its compactness and its separation margin, is directly and mathematically tied to the model’s ability to minimize the training loss, providing a strong theoretical justification for our approach.

The primary goal is to learn a latent space where distances directly reflect text‐pair similarity, transforming pre-trained word embeddings into a cluster-aware text representations. Furthermore, by maximizing inter‐cluster distances and minimizing intra‐cluster distances, this space mitigates overlap and enhances clustering performance, especially for short texts with limited context. This is particularly useful for extracting rich features and generating cluster-aware representations for short text that has limited contextual information.

In the following sections, we will explain about details of the internal structure of the proposed DSN-STC, which is designed to extract a rich feature pool and generate text representations based on these extracted features and their corresponding clusters.

#### Recurrent layer: Sequential features and long-term dependencies.

Recurrent layers are the sort of neural network that can help with sequential data and are therefore more geared towards these context-heavy domains of use, such as natural language processing and time series analysis. Unlike traditional neural networks, which treat inputs independently of each other, recurrent layers have loop connections that allow thoughts and memories to pass from one step to another. This preserved data from input helps the model to remember existing orders between sequences and works well in NLP fields like language modeling, speech recognition, and sequence prediction. But still, the vanilla recurrent layer has some problems which the most important of them is that as the sequence gets longer, the model cannot remember previous information. To overcome the shortcomings of vanilla recurrent layer, researchers developed more advanced models like Long Short-Term Memory (LSTM) [27] and Gated Recurrent Units (GRUs) [28]. These networks have modified structure that help them remember information for longer sequences and make them better suited for handling complex tasks.

In the proposed Siamese network, a recurrent layer is used to effectively capture the complex long-range dependencies between words and model sequential features. As discussed earlier, recurrent layers excel at handling sequential data, making them particularly well-suited for language tasks. By using a recurrent layer, the flow of text is modeled, capturing not only individual word meanings, but also how they interact and existing relations in sequence. By using a recurrent layer in the proposed network, it is ensured that the model can dynamically learn from the sequential flow of the text and improve its ability to represent complex patterns and relationships. In better words, we use a recurrent layer to extract sequential data and generate an informative representation at first as our first set of features in this network. As there are various types of recurrent layers, each was implemented and evaluated to determine which extracts the richest features for clustering. The evaluation results will be discussed in the next section.

#### Convolutional layers: Local features and N-grams dependencies.

Convolutional neural networks have gained significant success in the field of NLP because of their ability to process and analyze textual data effectively. While they were originally proposed and designed for image processing, they have been adapted for text by considering words or n-grams as spatial features, allowing these networks to extract and learn hierarchical features. This adaption enables convolutional layers to extract local features and relationships that lead to have good performance in NLP tasks such as text classification and categorization. As aforementioned earlier, one of the key advantages of using convolutional layers for processing textual data is the ability of these networks to model and extract existing local patterns between n-grams by applying convolutional filters on them. Extracting these sets of features can help to model the overall meaning of the text. This helps the model understand the meaning of the text in a way that traditional methods, which simply count words without considering their order or relationships, cannot [29]. In addition, convolutional layers can handle dimensionality challenges by using Pooling layers to reduce dimensionality in a way that the main and essential features preserves. This is especially useful for NLP tasks that involve input sequences of varying lengths [30].

Three convolutional layers were used as the second component of the hybrid feature extraction architecture. The core of this component consists of three parallel convolutional layers that each of them uses a distinct kernel size of 3, 5, and 7, respectively. The main purpose of this multi-scale approach is rooted in the linguistic diversity of short texts. Key information can be encoded in phrases of varying lengths, and a single kernel size would inevitably overlook critical patterns. Using linguistic examples from Persian, we can illustrate the necessity of this approach:

- A small kernel (size 3) is effective at capturing tight, meaningful collocations that act as a single semantic unit, such as «بورس اوراق بهادار» (‘stock exchange’).
- A medium kernel (size 5) can encompass a more complete event or short phrasal topic, like «افزایش قیمت سکه در بازار» (‘increase in coin price in the market’).
- A larger kernel (size 7) is necessary to capture longer-range dependencies within a single clause, where the key relationship spans several words, such as in «بانکی را اعلام کرد بانک مرکزی نرخ بهره بین» (‘The Central Bank announced the interbank interest rate’).

These general examples are representative of the n-gram structures found within used dataset [31], where key topics are often distinguished by such phrases of varying lengths. By using these kernels in parallel, the model can simultaneously detect these different types of features-from atomic entities to complete phrasal events- and create a richer and more robust representation for each short text. After each convolutional layer, a Pooling layer is used. These layers have two main responsibilities:

1) Dimensionality reduction that helps to computational cost efficiency
2) Translation invariance, enhancing the model’s robustness to variations in word positions. In other words, Pooling layers reduce the spatial dimensions of the feature maps generated by convolutional layers which causes the model to focus on the most salient features which not considering the minor variations in input. This characteristic is particularly beneficial in NLP, where the same meaning can be conveyed through different word arrangements [30].

In the last step, the feature vectors generated by each network are concatenated together. This concatenation process leads to generating a single comprehensive vector for each input short text, that represent a multi-scale encapsulation of features from different contextual windows.

This feature extracting process is design to complement the recurrent component of the proposed architecture which is described in Recurrent Path subsection. In other words, while the recurrent component is used for extracting sequential dependencies, the convolutional component focuses on extracting local features and patterns between n-gram. This approach will allow us to create a rich feature set for generating the final representations.

#### Fully connected layers: Feature concatenation and dimensionality reduction.

The third component of the proposed hybrid feature extraction architecture consists of several fully connected (FC) layers. These layers have an important role in efficiently integrating and refining features. The main characteristics of these layers can be explained in the following points:

1) Concatenation of feature vectors: These FC layers serve to fuse the rich feature sets that are extracted in previous components. This fused representation integrates sequential-based features extracted by recurrent component and local n-gram dependencies extracted by convolutional component.
2) Efficient dimensionality reduction: During feature fusion, these layers progressively reduce the dimensionality of the fused vector. This process is performed in a learned manner as opposed to a direct way which can cause the loss of important features. In other words, by learning to fuse and compress features rather than applying a direct reduction, the model can preserve the most salient information while yielding a dense final representation.

### Clustering text

Upon completion of training, the Text Embedding Encoder learns a cluster‐aware mapping from word embeddings into text representation and becomes a robust representation generator for input texts. It can then be applied to any short text to produce high‐quality, cluster‐discriminative representations. By emphasizing features that distinguish each cluster, the encoder enhances intra‐cluster cohesion and enlarges inter‐cluster separation, thereby improving overall clustering performance. In the subsequent clustering phase, these representations are supplied to a clustering algorithm (which is K-means in our implementation) to partition the texts into clusters.

### Experiments

This section presents the empirical evaluation of the proposed DSN-STC model. We first describe the experimental setup (datasets, pre-processing, and evaluation metrics) and the implementation details, then report the results of several experiments designed to evaluate model performance, justify architectural choices, and compare DSN-STC against state-of-the-art baselines.

### Experimental setup

All experiments were implemented in Python using the TensorFlow framework [32]. The DSN-STC model was trained using the hyperparameter settings detailed in Table 1, which were kept consistent across all relevant experiments unless otherwise specified.

The final clustering of the learned embeddings was performed using the K-means algorithm, as implemented in the Scikit-learn library. For evaluation purposes, the number of clusters, K, was set to the number of ground-truth classes in each dataset. While the standard K-means algorithm minimizes squared Euclidean distance, our model’s contrastive loss function operates on cosine distance. To ensure theoretical consistency between the training objective and the clustering metric, a critical normalization step was employed. During training, the cosine similarity calculation itself involves L2-normalizing the feature vectors (ensuring each has a Euclidean norm of 1) before comparison. Correspondingly, after the embeddings were generated from the trained model, we applied an explicit L2 normalization step to all embedding vectors (ensuring each vector has a Euclidean norm of 1) before feeding them to the K-means algorithm. This projects all embeddings onto a unit hypersphere, a space where Euclidean distance becomes a monotonic function of cosine distance. Consequently, the rank-ordering of distances is preserved, and minimizing one metric is equivalent to minimizing the other, thus formally aligning the clustering process with the learning objective.

### Dataset

As our main goal in this paper is short text clustering in the Persian language we used the Sep_TD_Tel01 dataset [31] which is a comprehensive Persian text collected from Telegram. This dataset was collected without specific restrictions such as keyword filtering that makes it a suitable sample of natural data stream from social media. This dataset has been used in other studies on the Persian language including NER [33], event detection [34], text clustering, and topic detection [35,36] which shows its usage and importance in Persian as a low-resource language.

In this dataset, for collecting data, a message collector system was developed at ComInSys (Computerized Intelligence Systems) lab that gathers text from all channels and groups. Approximately 23% of the messages were assigned topic labels, originally covering 75 distinct clusters which these data were used. After applying constraints that limited texts to 10–30 tokens, the number of clusters was reduced to 44. The Sep_TD_Tel01 dataset was randomly split into train, validation, and test (the ratio is reported at Table 1) using *random_seed = 73*. This seed controlled both data shuffling and model initialization for all experiments. In addition, more detailed information about the dataset can be found in Table 2.

**Table 2:** Summary of Dataset [31].

Parameter	Value
Number of posts	10,209
Number of super-Topics	2
Number of sub-Topics	81
Number of labeled posts	2,365

#### Data pre-processing.

**Fig 2:**
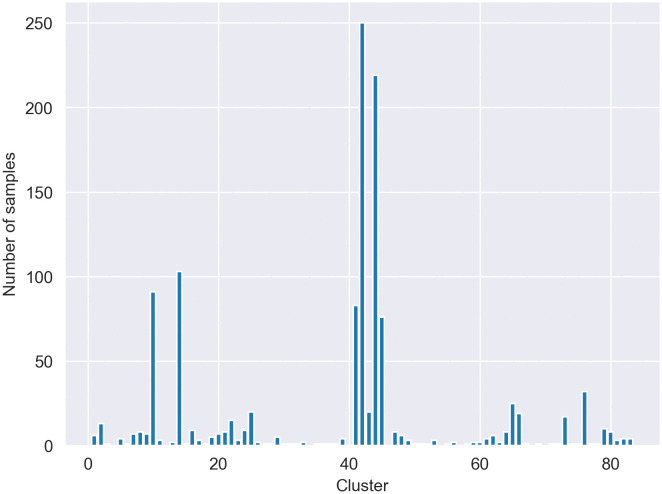
Tweet counts per cluster before pre-processing.

**Fig 3:**
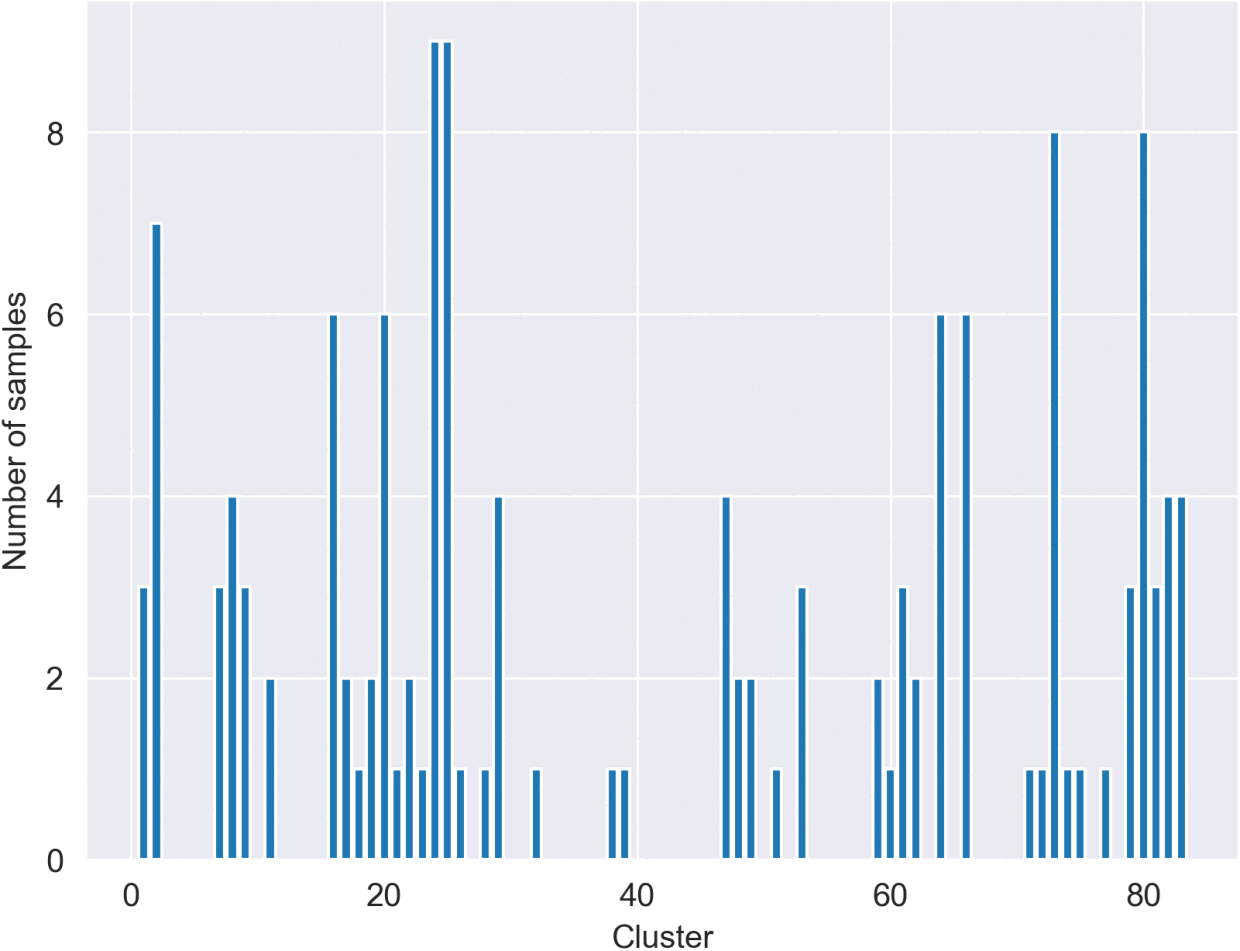
Tweet counts per cluster after pre-processing.

For pre-processing text in the dataset, as they contain a variety of noise like typos, emojis, URLs, and other artifacts, several pre-processing techniques [37] were used to remove these unnecessary elements from text and clean them while preserving informative content. The specific steps included text normalization, such as converting any embedded English/Latin characters to lowercase, and noise removal, where URLs, HTML tags, user mentions, and emojis were removed. To preserve the original signal, no stop-word removal or spell correction was performed. Also for tokenization, we used a custom tokenizer developed at the ComInSys lab, which is specifically designed for the morphological nuances of the Persian language. This pre-processing helps to preserve informative content. As explained earlier, token-length constraints are applied to text that leads to deleting those that have less than 10 or more than 30 tokens. Figs 2 and 3 show the number of tweets and their respective after preprocessing and normalization steps. After pre-processing, the resulting clean texts served as the basis for generating the different input representations used in this study. For the experiments utilizing TF-IDF, vectors were generated using a vectorizer configured with sublinear term frequency scaling (*sublinear_tf = True*) and a vocabulary limited to the top 300 most frequent features (*max_features = 300*). Min-max normalization is then performed on these embeddings to reduce the variance. This normalization helps accelerate training and improves convergence by scaling the data to a common range. The resulting embeddings are fed into the model as input.

### Evaluation metrics

Two metrics are used to evaluate the proposed method and compare it with other approaches in the text clustering field: Unsupervised Clustering Accuracy (ACC) and Normalized Mutual Information (NMI).

1 Unsupervised clustering accuracy (ACC): This metric measures the correspondence between assigned clusters and ground-truth cluster labels. The ACC formally is defined as follows:

$$\begin{array}{c} ACC=\text{max}(\frac{\text{1}}{N}\sum {}_{i=\text{1}}^{N}\delta \left(y_{i}=map\left({\hat{y}}_{i}\right)\right)) \end{array}$$ (6)

Where:

- ***N*** is the total number of data points
- ***y***_***i***_ is the true cluster label of the *i-th* data point.
- ${\hat{𝐲}}_{𝐢}$ is the assigned cluster to the *i-th* data point
- ***δ(x,y)*** is an indicator function equaling 1 if x = y and 0 otherwise
- $map({\hat{y}}_{i})$ illustrates a permutation function that maps each assigned cluster label ${\hat{𝐲}}_{𝐢}$ to the equivalent true cluster label using the Hungarian algorithm [38].

2 Normalized mutual information (NMI): For label set T and cluster set C, NMI is defined as:

$$NMI(K,P)=\frac{MI(K,P)}{\sqrt{E(K)E(P)}}$$ (7)

Where:

- ***MI(K, P)*** is the mutual information among K and P
- **E(K)** and **E(P)** is the entropies of K and P respectively.
- $\sqrt{E\left(K\right)E\left(P\right)}$ is used for normalizing the MI(K, P) to be in range [0, 1]

### Experimental results

#### Experiment1: Performance of different recurrent layers in the DSN-STC.

In this experiment, we aim to determine the most effective layer for the recurrent component of the proposed architecture. To this end, different types of recurrent layers including Long Short-Term Memory (LSTM), Gated Recurrent Unit (GRU), Bidirectional LSTM (Bi-LSTM), and Bidirectional GRU (Bi-GRU) were implemented and to determine which of these layers can perform better in our short text clustering context. The comparative results are presented in Table 3:

**Table 3:** Comparative Results of Various Recurrent Layers in the Recurrent Component of the DSN-STC.

Method	Type of used recurrent layer	Train		Test	
		ACC	NMI	ACC	NMI
DSN-STC	LSTM	0.74893	0.9078	0.74302	0.90844
	Bi-LSTM	**0.7681**	**0.9208**	**0.7669**	**0.9207**
	GRU	0.71786	0.88677	0.71886	0.88561
	Bi-GRU	0.74381	0.90714	0.73502	0.90575

The results presented in Table 3 indicate that the Bi-LSTM layer is the most suitable choice for our recurrent component. By processing each input sequence in both forward and reverse directions, the Bi-LSTM effectively doubles the contextual window available at every time step, enabling the hidden state to incorporate information from both preceding and succeeding tokens. From a theoretical standpoint, this bidirectionality enhances the representational capacity of the recurrent subspace: under the framework of sequence modeling, combining forward and backward state vectors increases the expressive power of the network, permitting it to approximate a broader class of sequence‐to‐representation functions. Furthermore, the gated architecture of the LSTM mitigates vanishing‐gradient issues, ensuring that long‐range dependencies (which is critical for disambiguating short texts) are retained in the learned state dynamics. These properties collectively yield richer, more nuanced feature sets, as necessary to resolve the inherent brevity and lexical ambiguity of short-text inputs. Accordingly, we adopt the Bi-LSTM as the recurrent module in our final DSN-STC configuration.

#### Experiment2: Effect of contrastive‐loss margin.

In Experiment 2, the margin hyperparameter *m* in Eq 2 was varied from 0.0 to 2.0 in steps of 0.1. For each margin value, DSN-STC was trained for 200 epochs with early stopping on validation loss (patience = 10). The resulting ACC and NMI were computed on both training and test splits and the final results are presented in Table 4.

**Table 4:** Comparative Results of Different Margin Levels in Contrastive Loss Function for Training the DSN-STC.

Margin levels	Train		Test	
	ACC	NMI	ACC	NMI
0	0.6597	0.8764	0.6513	0.8743
0.1	0.6579	0.8672	0.6577	0.8663
0.2	0.6930	0.8721	0.7048	0.8756
0.3	0.6929	0.8824	0.6993	0.8834
0.4	0.6841	0.8412	0.6974	0.8465
0.5	0.6548	0.8348	0.6588	0.8363
0.6	0.6438	0.8655	0.6510	0.8648
0.7	0.6791	0.8710	0.6926	0.8719
0.8	0.6766	0.8565	0.6894	0.8577
0.9	0.5157	0.7791	0.5317	0.7843
1.0	0.6267	0.8387	0.6518	0.8472
1.1	0.5693	0.8294	0.5805	0.8314
1.2	0.6868	0.8725	0.6910	0.8730
1.3	0.6068	0.8602	0.6060	0.8640
1.4	0.6518	0.8332	0.6441	0.8310
1.5	0.6667	0.8547	0.6636	0.8536
1.6	0.6651	0.8665	0.6734	0.8680
1.7	**0.7681**	**0.9208**	**0.7669**	**0.9207**
1.8	0.7234	0.8997	0.7286	0.8973
1.9	0.7069	0.8798	0.7176	0.8841
2.0	0.5977	0.8295	0.6127	0.8354

**Fig 4:**
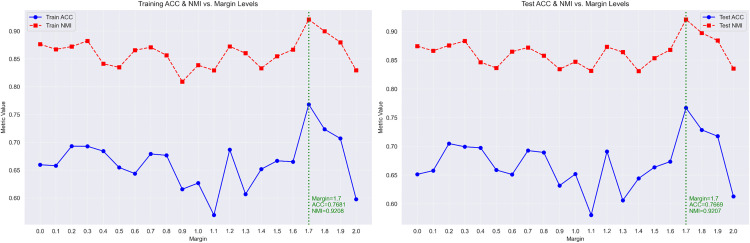
Evaluation of ACC and NMI as functions of the contrastive‐loss margin.

A clear peak in performance was found when the margin m was set to 1.7–1.8 (train ACC = 0.7681; test ACC = 0.7669; train NMI = 0.9208; test NMI = 0.9207). Performance degrades substantially for margins at the extremes: when *m ≤ 0.5*, clusters remain insufficiently separated, yielding low ACC and NMI (under-separation), and when *m ≥ 1.9*, the enforced separation becomes too strict, causing gradients to vanish and performance to drop (over-separation). Margins in the intermediate range *0.6 ≤ m ≤ 1.6* produce only moderate gains, indicating partial cluster separation. In the contrastive loss framework [39], setting the margin *m* too small results in insufficient separation between dissimilar pairs, as only distances within *m* incur a penalty. Conversely, an excessively large margin causes most dissimilar pairs to lie beyond *m*, ceasing to provide gradient updates for optimization. Therefore for all other experiments, we used m = 1.7. In addition, Fig 4 plots the effect of the contrastive margin *m* on clustering performance (ACC and NMI) and complements Table 4 by visualizing the margin sweep. It highlights the region around *m = 1.7* where both ACC and NMI peak and supports our selection of m = 1.7 for subsequent experiments.

#### Experiment3: Comparative clustering performance on the persian Sep_TD_Tel01 dataset.

To establish a robust set of state-of-the-art baselines for our comparative analysis, we selected several leading Sentence Transformer (SBERT) models. Given our focus on the Persian language, we specifically chose widely-adopted multilingual variants to ensure broad language coverage and provide a challenging benchmark. All models were sourced from the HuggingFace repository which is a comprehensive resource for standardized NLP models. The specific architectures, underlying pre-trained language models, and output embedding dimensions for each of the evaluated SBERT variants are detailed in Table 5.

**Table 5:** Details of sentence transformer models.

Base model	Sentence transformer model name	Output dimension
BERT (LaBSE-based)	use-cmlm-multilingual	768
Universal Sentence Encoder variant	distiluse-base-multilingual-cased-v2	512
MiniLM	paraphrase-multilingual-MiniLM-L12-v2	384
MPNet	paraphrase-multilingual-mpnet-base-v2	768
XLM-R	stsb-xlm-r-multilingual	768

A comprehensive evaluation of the proposed DSN-STC model was conducted on the Persian Sep_TD_Tel01 dataset. Following token-length filtering during preprocessing, DSN-STC (with margin = 1.7) was compared against both standard and new clustering techniques. For the standard baselines, each was evaluated using four word embedding schemes (TF-IDF, GloVe [40], FastText [41], and ParsBert [42]), while recent deep-clustering algorithms were assessed exclusively using ParsBert embeddings, which are pre-trained on Persian text. Clustering performance was quantified via ACC and NMI and evaluated both before and after token-length filtering to show the effect of the proposed constraint. The results of these comparisons are reported in Table 6.

**Table 6:** Clustering Performance (ACC, NMI) of Various Implemented Methods on the Sep_TD_Tel01 Dataset, Before and After Token-Length Filtering.

Method		ACC		NMI	
		Before Constraint	After Constraint	Before Constraint	After Constraint
K-means + TF-IDF		0.22071	0.36787	0.54651	0.69411
K-means + GloVe		0.21934	0.35924	0.55930	0.69379
K-means + FastText		0.18461	0.34728	0.52732	0.68137
K-means + ParsBert		0.38572	0.44911	0.65961	0.70192
DEC [7] + TF-IDF		0.22467	0.60162	0.59879	0.75949
DEC + GloVe		0.22561	0.59191	0.59619	0.73721
DEC + FastText		0.20371	0.48532	0.55192	0.74021
DEC + ParsBert		0.39271	0.62291	0.67174	0.77471
IDEC [9] + TF-IDF		0.24987	0.61013	0.58025	0.76537
IDEC + GloVe		0.24362	0.60928	0.60014	0.76482
IDEC + FastText		0.21928	0.5492	0.56372	0.69937
IDEC + ParsBert		0.39383	0.64928	0.68918	0.78283
AE with RN [13] + TF-IDF		0.28889	0.57314	0.57134	0.75276
AE with RN + GloVe		0.32725	0.61392	0.59172	0.78292
AE with RN + FastText		0.31371	0.48948	0.57834	0.76034
AE with RN + ParsBert		0.40837	0.61082	0.61928	0.83481
Stacked AE + ParsBert [43]		0.43328	0.71531	0.63014	0.88361
SBert [26]	use-cmlm-multilingual	0.3813	0.60149	0.59201	0.81583
	distiluse-base-multilingual-cased-v2	0.37541	0.59198	0.57568	0.81212
	paraphrase-multilingual-MiniLM-L12-v2	0.31355	0.59871	0.52849	0.79207
	paraphrase-multilingual-mpnet-base-v2	0.34595	0.57726	0.58475	0.79256
	stsb-xlm-r-multilingual	0.33711	0.58149	0.58838	0.80441
DAEC + ParsBert [44]		0.44713	0.74091	0.63921	0.90192
DSN-STC + TF-IDF		0.43928	0.7320	0.62973	0.8930
DSN-STC + GloVe		0.42883	0.71172	0.62628	0.86782
DSN-STC + FastText		0.40286	0.69492	0.61824	0.86265
DSN-STC + ParsBert		**0.46044**	**0.7669**	**0.65814**	**0.9207**

As shown in Table 6, imposing the token‐length constraint yielded substantial improvements across all methods, with average absolute gains of approximately 0.23 in ACC and 0.22 in NMI. One of our key objectives from this experiment is to see if this constraint can improve the clustering performance or not. Although DSN-STC + ParsBert already outperformed competing approaches even before filtering, this experiment confirms our first hypothesis: by excluding outlier texts that lack adequate context, clustering coherence is enhanced and overlaps are reduced, leading to marked performance gains for every methods tested.

Furthermore, our second hypothesis involved enabling the model to learn a transformation of pre-trained word embeddings into cluster-aware text representation, such that representations in this new latent space can be clustered more easily and accurately than in the original space. Indeed, after applying our Siamese constraint, DSN-STC + ParsBert achieved the best scores (ACC = 0.7669; NMI = 0.9207). In other words, by optimizing representations for both cluster membership and pairwise similarity, embeddings are mapped into a cluster-aware latent space where detection is significantly more accurate.

In addition to further contextualize the performance of DSN-STC, we established a strong state-of-the-art baseline by clustering sentence embeddings generated from several pre-trained multilingual SBERT models. The best-performing variant, *use-cmlm-multilingual + K-means*, achieved a test Accuracy of 0.60149 and a test NMI of 0.81583 (Table 6). This comparison is highly instructive and reveals a dual advantage of our proposed architecture. First, while SBERT embeddings are powerful, they are designed for general-purpose semantic representation and are not inherently optimized for the specific cluster structure of a downstream task. Second, and critically for a low-resource language, these multilingual models exhibit a known weakness on morphologically rich languages like Persian. Their shared BPE vocabularies frequently split Persian morphemes across subword boundaries, which can obscure root forms and impede the learning of coherent representations for nuanced constructs, a challenge noted in prior work [45]. In contrast, DSN-STC is architected to address both deficits. The substantial performance gap between the SBERT baseline and our DSN-STC + ParsBert model empirically demonstrates the primary contribution of our work. It confirms that our architecture’s advancement stems not only from its contrastive objective successfully learning a cluster-aware latent space, but also from its ability to leverage a language-specialized encoder (ParsBert) effectively and overcome the tokenization and representational challenges that general-purpose multilingual models face.

**Fig 5:**
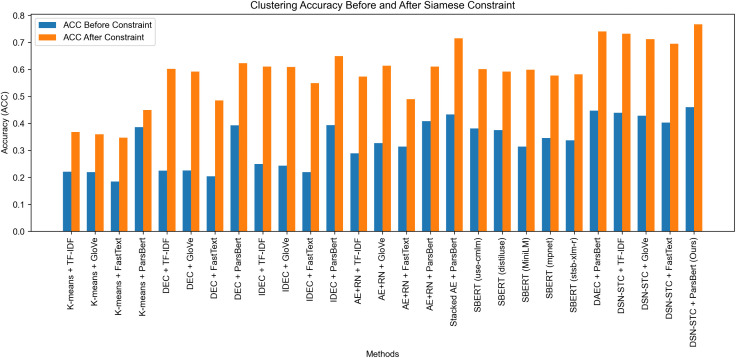
Comparison of ACC Metric Before and After Applying Constraints Across Different Methods.

**Fig 6:**
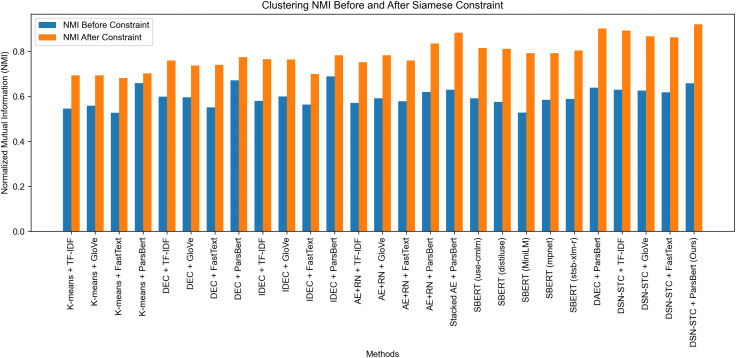
Comparison of NMI Metric Before and After Applying Constraints Across Different Methods.

The effectiveness of the token-length constraint and the overall superiority of our model are demonstrated in the results from Experiment 3, which are visualized in Figs 5 and 6. These figures display paired bar charts of ACC and NMI for each method, illustrating the results before and after applying the constraint. Consistent improvements in both metrics were observed across all embedding techniques following filtering constraint. Notably, DSN-STC + ParsBert achieved the largest improvements, with ACC increasing from 0.4604 to 0.7669 and NMI from 0.6581 to 0.9207. Also, these figures also allow for a direct comparison against strong transformer-based baselines. Even the best-performing SBERT model (*use-cmlm-multilingual*) achieved a final test accuracy of only 0.60149, substantially underperforming all variants of our DSN-STC model.

Importantly, DSN-STC outperformed competing methods within each embedding technique, underscoring its robustness in learning effective mapping from word embeddings into text representations. However, the intrinsic quality of those embeddings still influences the final latent space: higher-quality original embeddings yield more powerful cluster-aware representations. The charts provide a concise visual summary of each method’s comparative performance before and after applying filtering constraint. Moreover, DSN-STC already led the field even before filtering, confirming that (1) token-length constraints enhance clustering by removing uninformative texts and (2) the DSN-STC architecture’s ability to learn cluster-aware representation facilitates more accurate cluster detection.

#### Experiment4: Cross-linguistic generalizability.

Although our main focus of this study is to propose a clustering architecture for the Persian language, we also evaluate the robustness of the proposed method on two English benchmark datasets that are commonly used in clustering approaches, including 20newsgroups [46] and AGnews. This experiment allows us to test and evaluate the generalizability and adaptability of our approach in other languages and prepare good comparative results with other models. Table 7 summarizes these results:

**Table 7:** Comparison of DSN-STC Performance (ACC, NMI) Against Various Reported Methods on Standard English Benchmark Datasets.

Method	Dataset	
	20 newsgroups	AGnews
K-means	ACC: 0.37630 NMI: 0.41189	ACC: 0.30573 NMI: 0.22274
Hybrid model using RNN and AE [47]	ACC: 0.3736 NMI: 0.5066	–
AE [14]	–	ACC: 0.6748 NMI: 0.3299
STN-GAE [14]	–	ACC: 0.5742 NMI: 0.2914
SCA-AE [14]	–	ACC: 0.6836 NMI: 0.3414
DSN-STC	ACC: **0.80409** NMI: **0.89414**	ACC: **0.75948** NMI: **0.68001**

As shown in Table 7, the proposed method consistently outperforms other clustering approaches on English datasets. This not only highlights the strength and flexibility of the proposed method but also shows the potential of it to improve clustering in other language contexts. The improvements gained can confirm this experiment’s hypothesis that our method can perform better on clustering text not only in Persian but also in other languages. The reason for this is the ability of our method to extract and create rich feature sets from the limited context of the text, which leads to constructing richer cluster-aware representations for text.

#### Experiment5: Component ablation study.

To understand the individual contributions of each architectural component within DSN-STC, we conducted an ablation study that systematically removes one component at a time and measures its impact on clustering performance. Table 8 reports each variant of DSN-STC’s ACC and NMI alongside their absolute decreases (ΔACC, ΔNMI) relative to the full DSN-STC model. In this ablation study we sought to answer the following research questions:

**Table 8:** Impact of Component Ablation on DSN-STC Performance (Sep_TD_Tel01).

Variant	ACC (full)	ACC (ablated)	ΔACC	NMI (full)	NMI (ablated)	ΔNMI
Full DSN-STC	0.7669	—	—	0.9207	—	—
– Recurrent branch removed	0.7669	0.6184	–0.1485	0.9207	0.7646	–0.1561
– Conv1D branch removed	0.7669	0.6270	–0.1399	0.9207	0.7856	–0.1351
– Conv kernels [3, 5, 7] → [3 only]	0.7669	0.5809	–0.186	0.9207	0.7476	–0.1731
– Contrastive loss → Binary CE loss	0.7669	0.7182	–0.0487	0.9207	0.8753	–0.0454

1. RQ1: Which feature-extraction branch (recurrent vs. convolutional) contributes more to overall clustering performance?
2. RQ2: How critical is the multi-scale nature of the convolutional branch (kernel sizes [3, 5, 7] vs. a single size)?
3. RQ3: To what extent does the contrastive-loss objective improve cluster separation compared to a standard binary-cross-entropy loss?

As it can be seen in Table 8, every removal causes a clear drop in performance:

1. RQ1 finding: Removing the recurrent branch yields ΔACC = –0.1642 and ΔNMI = –0.1614, while removing the Conv1D branch yields ΔACC = –0.1556 and ΔNMI = –0.1504. Both pathways contribute nearly equally, indicating that sequence modeling and local pattern extraction each provide necessary information. The slightly larger impact of dropping the recurrent branch suggests that long-range dependencies carry marginally more weight in distinguishing clusters of short text.
2. RQ2 finding: Restricting the convolutional branch to a single kernel size incurs the largest drop (ΔACC = –0.2017; ΔNMI = –0.1784). This result empirically confirms our architectural motivation. Multi-scale convolutions capture patterns at varying n-gram windows, from short phrases to longer collocations. Removing this diversity forces the model to overlook certain granularities of meaning; for instance, a model with only a small kernel might identify key entities but fail to capture the broader phrasal context that defines a cluster’s topic, as explained by the linguistic examples in the Methods section. This shows that capturing features across multiple scales is crucial for representing short texts effectively. In other words, giving the model the ability to capture local dependencies in different windows allows it to extract much more discriminative features that can find and highlight the important phrases, helping the model to better model the subject of each text and generate final representations more accurately.
3. RQ3 finding: Replacing contrastive loss with binary cross-entropy still degrades performance (ΔACC = –0.0644; ΔNMI = –0.0807). While binary cross-entropy still enforces some degree of separation, its relatively smaller ΔACC compared to the other ablations indicates that architectural components (recurrent + multi-scale conv) can partially structure the embedding space well. However, the contrastive objective’s explicit push–pull mechanism remains essential for maximizing inter-cluster margins and minimizing intra-cluster variance, delivering the highest cluster cohesion.

These results confirm that each component of DSN-STC is vital for producing high-quality, cluster-aware representations.

#### Experiment6: Hyperparameter sensitivity analysis.

To further validate our architectural choices and assess the model’s stability, we conducted a sensitivity analysis on three key hyperparameters: the number of units in the recurrent layer, the combination of kernel sizes in the multi-scale convolutional branch, and the number of filters in each convolutional layer. In these experiments, we varied one hyperparameter at a time while keeping all others fixed to their optimal values as listed in Table 6. The comprehensive results of this analysis are presented in Table 9.

**Table 9:** Sensitivity Analysis of Key Hyperparameters for DSN-STC.

Hyperparameter	Value	Train Accuracy	Test Accuracy	Train NMI	Test NMI
Recurrent Units	100	0.759	0.759	0.905	0.906
	200	0.7681	0.7669	0.9208	0.9207
	300	0.728	0.739	0.866	0.868
Kernel Sizes	[2, 3, 4]	0.614	0.614	0.813	0.814
	[2, 4, 6]	0.710	0.704	0.882	0.880
	[3, 5, 7]	0.7681	0.7669	0.9208	0.9207
	[5, 7, 9]	0.666	0.672	0.877	0.877
Conv1D filters	32	0.659	0.651	0.863	0.860
	64	0.7681	0.7669	0.9208	0.9207
	128	0.614	0.615	0.799	0.800

The results provide several key insights into the model’s behavior. First, for the Recurrent Units, we observe a clear performance peak at our chosen value of 200. While 100 units also perform well, increasing the capacity to 300 units leads to a notable degradation in test performance (from 0.7669 to 0.739 ACC), suggesting the onset of overfitting and confirming that 200 units provides a robust balance between representational power and generalization.

Second, the analysis of Kernel Sizes empirically validates our multi-scale design hypothesis. The [3, 5, 7] configuration significantly outperforms all other variants. The underperformance of configurations focused on exclusively smaller ([2, 3, 4]) or larger ([5, 7, 9]) n-grams indicates that the model must capture patterns across a balanced spectrum of linguistic scales, from tight collocations to longer phrases, to effectively represent the short texts in our dataset.

Finally, the sensitivity to the number of Conv1D filters highlights the importance of model capacity. Using 32 filters provides insufficient representational power, leading to lower performance. Conversely, increasing the filter count to 128 causes a sharp drop in both Test Accuracy and NMI. This is a classic indicator of overfitting, where the overly complex convolutional branch begins to model noise rather than generalizable features, validating our choice of 64 filters as optimal.

Overall, this sensitivity analysis demonstrates that the hyperparameters chosen for DSN-STC are not arbitrary but are located within a stable and high-performing region of the parameter space, confirming the robustness of our model’s architecture.

#### Experiment7: Computational cost analysis.

To evaluate the practical viability and scalability of our proposed model, we conducted an analysis of its computational cost, focusing on training time. For a fair comparison, all experiments were conducted on a Google Colab instance equipped with a 15 GB NVIDIA T4 GPU and 12 GB of RAM. We measured the training time for our full DSN-STC model and compared it against its ablated variants as well as key transformer-based baselines. The results, presented in Table 10, provide two key insights that address both the internal cost-benefit of our hybrid design and its external comparison to baselines.

**Table 10:** Training time and performance comparison for DSN-STC and key baselines.

Model	Test Accuracy	Test NMI	Training Time (seconds)
ParsBert + K-means	0.44911	0.70192	0.154
SBERT + KMeans	0.60149	0.81583	30.570
DSN-STC (Recurrent branch only)	0.6270	0.7856	270.39
DSN-STC (Conv1D branch only)	0.6184	0.7646	114.86
DSN-STC (Full Model)	0.7669	0.9207	846.38

First, the analysis of our model’s components is highly informative. The recurrent-only branch (270.39s) is substantially more computationally expensive than the convolutional-only branch (114.86s), which is expected due to the sequential nature of recurrent operations. While the full hybrid model (846.38s) requires the most training time, our ablation study (Experiment 5) has already demonstrated that both branches are essential for achieving optimal clustering performance. This confirms that the combination of these complementary feature extractors is an effective use of computational resources.

Second, when compared against the strong baselines, DSN-STC demonstrates a highly favorable performance-to-cost trade-off. While our model is naturally more computationally intensive than direct clustering on pre-computed embeddings, this increased cost yields a massive improvement in clustering quality—a gain of over 0.16 in Test Accuracy compared to the best SBERT baseline. This substantial performance leap validates our end-to-end, cluster-aware training approach. It confirms that DSN-STC provides a state-of-the-art accuracy advantage for a reasonable and justifiable increase in computational cost, making it a viable and effective solution.

#### Experiment8: Statistical validation of DSN-STC improvements.

To rigorously assess whether DSN-STC’s performance gains are statistically reliable, paired *t*-tests (two-tailed, α = 0.05) were conducted on *N* = 10 independent runs for both ACC and NMI. Two sets of comparisons were evaluated:

1. **Token-Length Constraint Impact:** DSN-STC trained on the full dataset versus with token-length filtering.
2. **Baseline Competitors:** DSN-STC (filtered) versus each of six baselines, all using ParsBert embeddings.

Table 11 summarizes the *t*-statistics and corresponding *p*-values for each comparison. All *p*-values fall below the 0.05 threshold, confirming that DSN-STC’s improvements in both ACC and NMI are statistically significant across preprocessing conditions and when compared to a broad spectrum of clustering methods.

**Table 11:** Paired t-test for DSN-STC performance (ACC and NMI) on the Sep_TD_Tel01 dataset.

Comparison	ACC Metric (t-test)		NMI Metric (t-test)	
	t-statistic	p-value	t-statistic	p-value
Before vs. After token-length constraint	9.991	0.0010	11.181	0.0010
DSN-STC vs. K-means	3.099	0.0127	5.961	0.0020
DSN-STC vs. DEC	2.601	0.0287	4.786	0.0010
DSN-STC vs. IDEC	2.095	0.0357	6.786	0.0010
DSN-STC vs. AE with RN	1.861	0.0368	5.310	0.0050
DSN-STC vs. Stacked AE	2.759	0.0421	2.250	0.0410
DSN-STC vs. DAEC	2.655	0.0462	2.310	0.0463

## Discussion

In this study, we propose a novel architecture for clustering short text that has a specialized Siamese network-based model. The key innovation of our approach lies in its ability to transform textual data from an initial word embedding space into a cluster-aware text representation latent space that is more efficient for clustering. This transformation that is learned within a Siamese network employing multi-scale hybrid feature extraction enables the model to cluster text data more effectively by generating representations that capture rich, cluster-aware features.

Several experiments were conducted to evaluate the effectiveness of our approach. In the first experiment, different recurrent neural layers were implemented and compared to see that which of them is the most suitable choice that can extract and model sequential dependencies and features better than others. The comparative experiment showed that the Bi-LSTM layer is the most suitable choice, outperforming other recurrent layers like LSTM, GRU, and Bi-GRU. This result underscores the importance of bidirectionality in extracting richer and more informative features from text sequences.

In Experiment 2, we performed a comprehensive margin sweep over the contrastive‐loss parameter m*∈ [0,2]*. We observed a characteristic unimodal performance curve, with insufficient separation at *m* ≤ *0.5*, excessive separation at *m* ≥ *1.9*, and an optimal peak at *m = 1.7* that confirms that balanced intra-cluster cohesion and inter-cluster separation are essential for maximizing ACC and NMI. In addition, future work could explore dynamic margin selection strategies to further adapt the contrastive objective during training.

In Experiment 3, we compared DSN-STC against both standard baselines and recent, advanced clustering approaches on the Sep_TD_Tel01 dataset. We focued on 2 main questions for this experiment: “Does applying token-length constraints improve clustering performance?” and “How does our method compare to previous approaches?”. As shown in Table 6, enforcing the token-length constraint yielded average gains of 31% in ACC and 27% in NMI. Notably, even before applying these constraints, DSN-STC delivered improvements of 1.3% in ACC and 2.7% in NMI over competing methods, which shows its intrinsic ability to extract rich, cluster-aware features.

These results demonstrate that DSN-STC effectively learns to map pre-trained word embeddings into a text representation latent space where cluster overlap is minimized and separability is maximized, thereby facilitating more efficient clustering. In addition, the superior performance of DSN-STC + ParsBert (ACC = 0.7669, NMI = 0.9207) derives from its capacity to fuse contextualized embeddings with multi-scale hybrid feature extraction. Unlike static embeddings (e.g., GloVe), ParsBert encodes nuanced semantic relationships in short texts, while our hybrid architecture preserves and combines both long-range dependencies and local n-gram patterns for enhanced cluster discrimination.

While our primary evaluation targeted Persian text, we also assessed the robustness of DSN-STC on standard English clustering benchmarks in the experiment 4. These cross-linguistic evaluations demonstrate that our architecture generalizes well beyond Persian, consistently outperforming established clustering methods. Importantly, these results underscore that the quality of the final cluster-aware latent space is intrinsically tied to the quality of the initial embedding space. This principle mirrors the findings of Moslem et al. [48], who generated synthetic bilingual terminology data with an LLM, fine-tuned a machine-translation model on that data, and then applied LLM-guided post-editing to enforce domain terms, nearly doubling term integration and demonstrating the power of domain-aware data augmentation for specialized tasks. Consequently, applying DSN-STC to different languages demands high-quality embedding techniques, especially contextualized models such as BERT or its language-specific variants, to ensure that the initial representations encode sufficient syntactic and semantic nuance. Indeed, Rezaei et al. [49]demonstrated across eight sentiment‐analysis benchmarks that the combination of deep architectures and carefully selected word embeddings can lead to up to a 15 point accuracy swing, further underscoring the downstream impact of embedding quality in diverse NLP tasks. Also, Wassie *et al.* recently showed in [50] that fine-tuning open-source large language models on domain-specific corpora yields substantial accuracy gains in specialized translation tasks, underscoring the value of domain-tuned contextual embeddings for high-fidelity representation learning. In practice, leveraging good embeddings for each target language is essential to realize the full potential of our Siamese contrastive framework and to achieve comparable performance gains across diverse linguistic contexts.

Experiment 5 (the ablation study) evaluated DSN-STC by removing each core component in turn, including recurrent branch, convolutional branch, multi-scale kernels, and the contrastive-loss head, and measured the resulting ΔACC and ΔNMI. Every deletion caused a substantial performance drop (up to –0.20 in ACC), confirming that all architectural elements are indispensable for generating high-quality, cluster-aware text representations.

Recurrent layers excel at modeling long-range dependencies and capturing topic coherence across a sequence, while convolutional layers efficiently detect salient n-gram patterns. Their complementary roles explain why removing either branch significantly degrades performance. Moreover, restricting the convolutional path to a single kernel size produced the largest decrease (ΔACC = –0.20), which highlights the necessity of multi-scale feature extraction. The reason is short texts often contain phrases of varying lengths, such as “climate change” (2-gram) versus “machine learning in healthcare” (4-gram) that multi-scale kernels can adapt to this variability and extract valuable features from diverse n-gram patterns, whereas a single kernel cannot capture such linguistic diversity.

Finally, replacing the contrastive loss with binary cross-entropy (BCE) resulted in a notable drop as well, underscoring that BCE’s independent treatment of pairs lacks the structural regularization provided by the contrastive objective, which explicitly enforces both intra-cluster compactness and inter-cluster margins. This dual push–pull mechanism is critical for learning text representations that faithfully reflect cluster structure. In addition, Experiment 6 employed paired t-tests (at *p < 0.05*) on ACC and NMI gains, confirming that all observed improvements, including token-length filtering and architectural design, are statistically significant and not due to chance.

In addition to previous core performance comparisons and ablation studies, our final experiments were designed to validate the robustness and practical viability of the DSN-STC architecture. The hyperparameter sensitivity analysis (Experiment 6) confirmed that our chosen configuration is not fragile; the selected values for recurrent units, kernel sizes, and filter counts reside within a stable performance peak, demonstrating that the model’s strong performance is a robust property of the architecture. Furthermore, the computational cost analysis (Experiment 7) provided a clear view of the performance-cost trade-offs. It confirmed that DSN-STC provides a state-of-the-art accuracy advantage for a justifiable increase in training time compared to other methods. It is important to note, however, that our exhaustive pairwise training strategy has a time complexity of $O\left(N^{\text{2}}\right)$ with respect to the number of training samples *N*. Consequently, while highly effective for datasets of the scale used in this study, applying DSN-STC to significantly larger corpora would likely require more sophisticated training pair construction to maintain computational cost efficiency. Taken together, these final analyses validate that DSN-STC is not only effective but also robust and computationally practical for its target application.

A further methodological consideration is the approach to model regularization. The contrastive loss function in Equation (3) does not include an explicit regularization term, such as L2 weight decay, which is often used to penalize model complexity. In this study, we instead relied primarily on Early Stopping (with a patience of 10 on the validation loss) as our primary mechanism for preventing overfitting. This technique is a powerful and widely used form of temporal regularization that halts the training process once generalization performance on unseen data no longer improves. By selecting the model at its optimal point in the training trajectory, Early Stopping implicitly prevents the network’s weights from becoming overly specialized to the training set, serving a similar goal to explicit weight decay. Furthermore, the Siamese architecture itself provides a form of structural regularization through its shared weights and encourages generalization. Also, while our results demonstrate that these methods were sufficient for achieving strong performance, the exploration of explicit weight decay could be a valuable direction for future work.

In the end, it is worth noting a key consideration regarding the scope of this work. While the proposed architecture outperforms other baseline and more advanced recent clustering architectures, the imbalanced nature of the Sep_TD_Tel01 dataset, as seen in Figs 2 and 3, may have influenced the results. Class imbalance and the sparsity of rare classes are known to complicate short-text clustering: minority classes are often under-represented and therefore harder to group reliably. Contrastive and mixup-style approaches have been proposed to mitigate such low-resource challenges [51]. More specifically, prior work has shown that class imbalance can bias algorithms toward majority classes and degrade minority-class recovery in both clustering and classification tasks, and that contrastive objectives may benefit from imbalance-aware modifications (e.g., weighted or asymmetric losses, resampling strategies) to avoid deteriorated performance on underrepresented classes [52,53]. Although some clusters in the used Persian dataset are relatively small, we did not apply class-imbalance mitigation (e.g., weighted loss or resampling) in this study because our primary goal was to validate the Siamese hybrid architecture.

In conclusion, the results of our experiments confirm the strength and adaptability of the proposed architecture in learning rich, cluster-aware mapping from word embeddings into text representations. Our model demonstrates both effectiveness and adaptability across different languages, providing a robust solution for short text clustering in diverse linguistic contexts.

## Conclusions and future works

In this work, we proposed a novel architecture DSN-STC, based on a Siamese network specifically designed to improve the clustering of Persian short text. Our main idea was to train a model that can transform word embeddings from an initial space which is generated by a pre-trained model, into cluster-aware text representations where clusters can be detected more easily and improve the clustering by minimizing the overall overlapping between existing clusters. In other words, the representations of text that are generated by the proposed architecture can capture both the structural and contextual features of each text as well as preserving and improving the cluster-relevant features. So that our model provides a more accurate representations for clustering tasks. The DSN-STC model employs a Siamese network with multi-scale hybrid architecture, consisting of one recurrent layer and three convolutional neural networks to extract both sequential and local features from the text respectively. These extracted features are then concatenated through fully connected layers to produce the final representation of the input text. During the training phase, the Siamese network takes pairs of inputs and then is trained based on the similarity of their cluster assignments, using a contrast loss function to update the model parameters. In better words, when two inputs belong to the same cluster, they are considered similar, and the model minimizes the distance between them. Conversely, for inputs from different clusters, the model maximizes their distance. This loss function, by minimizing the distances for similar data and maximizing those for dissimilar data, enables our model to learn high-quality, cluster-aware representations that are well-suited for short text clustering. In fact, each short text within its limited context inherently includes sequential and long-range dependencies and also local n-gram patterns in different windows. By the proposed multi-scale hybrid feature extraction architecture, The proposed multi-scale hybrid feature extractor is designed to maximize the extractable diverse features to highlight the main subject of the text and then give ability to the model to learn which complementary feature subsets it should use to generate the final text representation based on each cluster. In this way model can learn a cluster-aware mapping from the initial word embedding space into a text representation latent space and improve the clustering performance. Our experiments show significant improvements in clustering accuracy and NMI metrics compared to previous methods. Although our main focus was on clustering Persian data, DSN-STC was also evaluated on commonly used English dataset, where it continued to show significant improvements over other methods. This cross-linguistic performance highlights the robustness and adaptability of our model for short text clustering tasks.

For future works, we suggest exploring additional pre-trained embedding models to determine whether they can further improve our model’s performance. Also using attention mechanisms in the architecture may provide further improvements in clustering because in this way, model can learn better to attend to which set of features and use them for generating the final representations. A particularly important direction, as noted in our discussion, is the investigation of imbalance-aware training strategies, such as weighted loss functions or resampling techniques, to potentially improve performance on minority clusters. Finally, while our study managed the inherent noise of the real-world dataset through pre-processing, a valuable future direction involves leveraging these signals instead of only removing them. Techniques such as incorporating dedicated emoji embeddings or applying robust spell-correction could potentially capture additional semantic cues and probably enhance clustering performance. These avenues present promising opportunities to build upon the strong foundation established by the DSN-STC model.
